# Preparation, optimization and swelling study of carboxymethyl sago starch (CMSS)–acid hydrogel

**DOI:** 10.1186/s13065-018-0500-8

**Published:** 2018-12-06

**Authors:** Nur Fattima’ Al-Zahara’ Tuan Mohamood, Norhazlin Zainuddin, Mansor Ahmad@Ayob, Sheau Wei Tan

**Affiliations:** 1 Department of Chemistry, Faculty of Science, Universiti Putra Malaysia, 43400 Serdang, Selangor Malaysia; 2 Laboratory of Vaccine and Immunotherapeutic, Institute of Bioscience, Universiti Putra Malaysia, 43400 Serdang, Selangor Malaysia

**Keywords:** Carboxymethyl sago starch, Optimization, Hydrogel, Gel content, Swelling in different media

## Abstract

In this study, sago starch was modified in order to enhance its physicochemical properties. Carboxymethylation was used to introduce a carboxymethyl group into a starch compound. The carboxymethyl sago starch (CMSS) was used to prepare smart hydrogel by adding acetic acid into the CMSS powder as the crosslinking agent. The degree of substitution of the CMSS obtained was 0.6410. The optimization was based on the gel content and degree of swelling of the hydrogel. In this research, four parameters were studied in order to optimize the formation of CMSS–acid hydrogel. The parameters were; CMSS concentration, acetic acid concentration, reaction time and reaction temperature. From the data analyzed, 76.69% of optimum gel content was obtained with 33.77 g/g of degree of swelling. Other than that, the swelling properties of CMSS–acid hydrogel in different media such as salt solution, different pH of phosphate buffer saline solution as well as acidic and alkaline solution were also investigated. The results showed that the CMSS–acid hydrogel swelled in both alkaline and salt solution, while in acidic or low pH solution, it tended to shrink and deswell. The production of the hydrogel as a smart material offers a lot of auspicious benefits in the future especially related to swelling behaviour and properties of the hydrogel in different types of media.

## Introduction

Sustainable chemistry is a green approach in science and technology for environmental protection where this approach is hoped to overcome serious issues related to the ecosystem. Researches on carbohydrates polymer have been actively done due to their sustainability and biodegradability properties. These biodegradable polymers such as starch [1], chitosan [2] and carrageenan [3] can simply be modified via crosslinking, cationization, UV-irradiation, microwave and electron beam irradiation [4]. In recent years, numerous types of technologies are used to improve the world’s climate which brings biodegradable technology as one of the examples that offers environmental solutions without harming the planet.

Starch is a natural polymer produced by green plants to store energy that can be easily found in leaves, stems, roots and seeds. Sago starch is isolated from the sago palm through the process of extraction and purification. Malaysia as world’s largest sago exporter has been exporting sago products in the volume of 44,000 tonne per year to Japan, Europe, America and Singapore [5]. Sago palm is produced commercially in Sarawak, where the crop is mainly grown on peat soils. The most common sago species grown is *Metroxylon sagu* because this type of sago plant gives higher quality products [6]. According to Flach [7], the advantages of the crops are; environmentally friendly, uniquely versatile and promote socially stable agroforestry systems. Plus, this crop is imperious to some minor natural disasters such as floods, drought, fire and strong winds because of its large fibrous root. Sago has been widely used around the world and the diversity has led to the use of sago in many areas.

The versatility of sago starch is due to its physicochemical properties that can easily be altered through chemical or physical treatment [8]. The modification of sago starch is crucial to intensify its industrial properties and these modifications have been reported to improve its swelling, solubility and light transmittance [9]. Modification by crosslinking can be established via chemical reaction that is initiated by the change in pH, radiation, heat or pressure [10]. Crosslinking treatment is performed to increase chemical bonds at random locations in a granule to make it stable and strengthen the relatively tender starch. According to Haroon et al. [11], the treated starch via crosslinking may embellish the tensile strength and thermal stability. Modified sago starch such as carboxymethyl sago starch (CMSS) is proclaimed to improve physicochemical properties such as swelling ability in cold water, freeze–thaw stability and low retrogradation tendency [12].

Hydrogel is a polymeric three-dimensional (3D) network gel that is formed by polymer chains crosslinking, composed of hydrophilic groups such as hydroxyl and carboxyl to store water and biological fluid. To ensure that the hydrogel is equipped with hydrophilic character, carboxylic acid groups (R-COOH) is needed as the side groups of the hydrogel backbone. The absorption capacity and swelling properties of this sensitive hydrogel are very important in most of its applications. The hydrogel has a potential to swell in different media, highly associated with the network porosity and depends on; crosslinking density and hydrogel-media attraction [13]. Hydrogel is an example of smart material because of its ability in changing structure due to certain responses. This smart hydrogel is able to change its volume in different environmental responses such as temperature, pH, ions and substances concentration [14]. Hundreds of hydrogels from natural polymer have been fabricated using starch, alginate and chitosan because of their potential application in biomaterial field due to their safety, hydrophilicity, biocompatibility and biodegradability. Year by year, researchers are doing their best to modify and improve the hydrogel properties so that its usage can be expanded and not limited only to certain areas.

In this research, sago starch was chosen to be modified due to its abundancy and low cost. The aim of this research was to optimize the preparation of CMSS–acid hydrogel and to study its swelling properties in different media.

## Materials and methods

### Materials

Sago starch powder was purchased from Song Ngeh Sago Sdn Bhd, Sarawak, Malaysia. Sodium monochloroacetate (SMCA, Sigma-Aldrich), sodium hydroxide (NaOH, ChemAR^®^) pellets, isopropanol (IPA), methanol, ethanol, acetic acid, phosphate buffer saline (PBS) solution pH 2.0, 7.4 and 10.0 were purchased from the R&M Chemicals. All chemicals used in the study were of analytical grade. Deionized and distilled water were used throughout the experiment.

### Preparation of CMSS

**Fig. 1:**
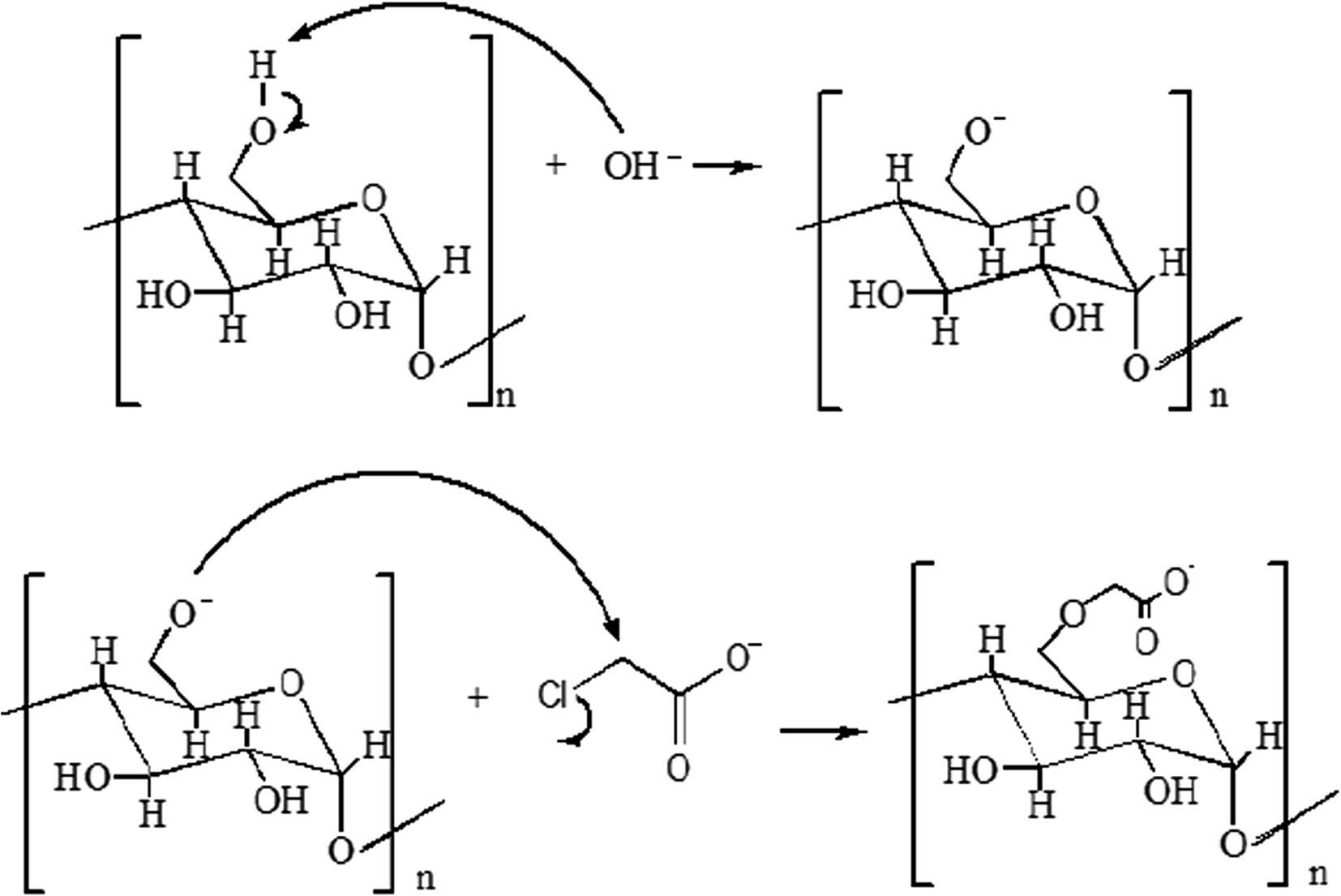
Schematic diagram of CMSS synthesis via Williamson ether synthesis using NaOH and SMCA

CMSS was prepared by following the method published by the previous study [15]. The sago starch was modified using carboxymethylation method. CMSS was prepared according to the Williamson ether synthesis (Fig. 1) by activation of sago starch with aqueous alkali hydroxide mostly NaOH and it is reacted with monochloroacetic acid or its sodium salt [16]. Purification of CMSS was done by washing the CMSS with ethanol (85% purity). This procedure was repeated three times.

### Determination of degree of substitution (DS)

The degree of substitution (DS) of carboxylic group in CMSS is defined by the average number of the hydroxyl group in the starch structure which was substituted by carboxymethyl groups. The DS of the sample was determined by the standard ASTM D1439 [17].

### Preparation of CMSS hydrogel

**Fig. 2:**
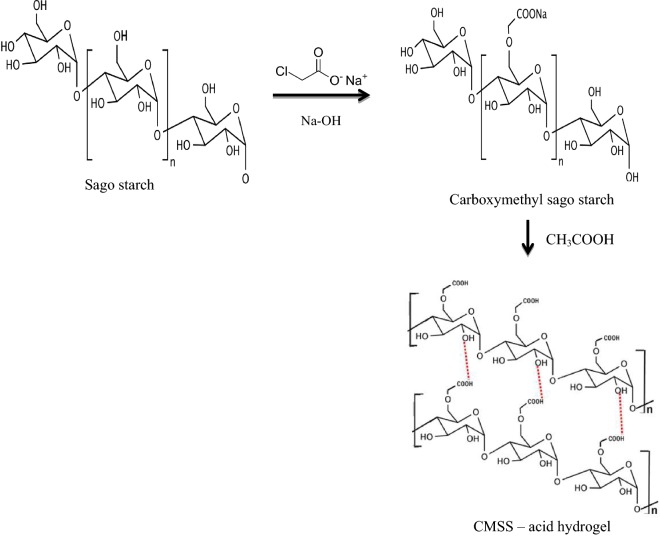
Diagrammatic scheme of sago starch, CMSS and CMSS–acid hydrogel

50–90% (w/v) of CMSS were dissolved in 2.0 M acetic acid. The pastes were placed in petri dish covered with parafilm and kept at room temperature for 24 h. A small amount of CMSS–acid hydrogel was taken for the determination of gel content and degree of swelling. The diagram of preparation of CMSS and CMSS–acid hydrogel is shown in Fig. 2. The optimization of the CMSS–acid hydrogel was studied by four parameters which were; (1) concentration of CMSS; (2) concentration of acetic acid; (3) reaction time and (4) reaction temperature.

The CMSS–acid hydrogel was purified with distilled water to remove the uncrosslinked CMSS and the excess of acetic acid. This purified CMSS–acid hydrogel was then sent for characterizations.
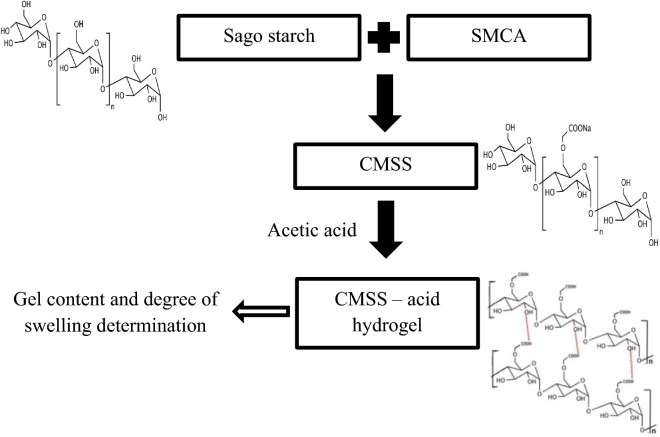


### Gel content and degree of swelling

CMSS–acid hydrogel was immersed in distilled water for 72 h at room temperature. Then, the hydrogel was placed in an oven at 70 °C for 72 h and the percentage of gel content was calculated using the formula:

1 $${\text{Percentage of gel content}} = {\text{W}}_{\text{ai}} /{\text{W}}_{\text{bf}} \times { 1}00$$

where W_ai_ is the weight of dried sample after immersion and W_bf_ is the weight of dried sample before immersion.

The swelling study of the CMSS–acid hydrogel was carried out in distilled water. The sample of CMSS–acid hydrogel was placed in teabag and immersed for 72 h at room temperature. After it had reached equilibrium, the hydrogel was weighed. The degree of swelling is calculated using the following formula:

2 $${\text{Degree of swelling }} = \, \left( {{\text{W}}_{\text{s}} {-}{\text{W}}_{\text{d}} } \right)/{\text{W}}_{\text{d}}$$

where W_s_ is the weight of swollen sample after immersion in distilled water and W_d_ is the weight of dried sample.

### Swelling test in different media

1.0 g of optimized CMSS–acid hydrogel was weighed into a teabag and immersed in a beaker of 150.0 mL of different medium for 72 h at room temperature. The media studied were; (1) 0.2 M of NaCl solution; (2) 0.5 M of NaCl solution; (3) 1.0 M of NaCl solution; (4) 1.0 M of NaOH solution; (5) 1.0 M of HCl solution; (6) PBS solution pH 2.0; (7) PBS solution pH 7.4 and (8) PBS solution pH 10.0. After the immersion, CMSS–acid hydrogel was weighed again and then, the degree of swelling was calculated using Eq. 2.

### Fourier transform-infrared spectroscopy (FT-IR)

FT-IR spectroscopy is a technique used to determine the functional groups of the sample by measuring the infrared absorption spectrum. FT-IR spectra were recorded on FT-IR spectrometer (Spectrum 100 Perkin Elmer) with a wavenumber range between 400 and 4000 cm^−1^. The sampling technique used was attenuated total reflection (ATR) in conjunction with infrared spectroscopy, which enables the samples to be examined directly in the solid state.

### X-ray diffraction (XRD)

X-ray diffraction is a technique used to reveal the information on the structure of a sample. This XRD characterization was carried out using Shimadzu XRD-6000 diffractometer with Cu Kα (λ = 1.5418 Å) radiation at room temperature operated at 30 kV and 30 mA. A sample was placed in an aluminium sample holder and a diffraction pattern plots intensity against the angle of the detector, 2θ and the scanning range 2° to 60° with rate of 2°/min with continuous scan mode.

### Scanning electron microscopy (SEM)

Scanning electron microscopy is a technique used to study the surface morphology of a material and it basically focuses on the surface of the material and its composition. The samples were freeze-dried first and then gold sputter-coated to make the samples become conductive before the scanning process is done. The prepared samples were examined under scanning electron microscope (JEOL, Tokyo Japan) at a voltage of 15.0 kV and recorded at the range of magnification between 50 and 1000×.

## Results and discussion

### Degree of substitution (DS)

The degree of substitution (DS) of CMSS attributes to the average number of carboxymethyl groups per anhydroglucose unit (AGU) and theoretically, the maximum number of DS is 3.0 [18]. In this study, the sago starch was modified via chemical modification using SMCA and the DS was found to be 0.6410.

### Effect of CMSS concentration

**Fig. 3:**
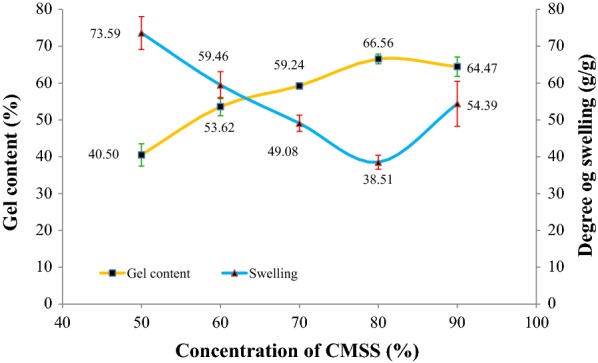
Effect of CMSS concentration on gel content and degree of swelling of CMSS–acid hydrogel

Gel content and degree of swelling of CMSS–acid hydrogel at different percentage of CMSS were shown in Fig. 3. The controlled variables for this parameter were molarity of acetic acid at 2.0 M, 24 h reaction time and 27 °C reaction temperature. A weak hydrogel paste was produced with CMSS concentration lower than 50% (w/v). A weak hydrogel is defined as a gel that dissolved in water which is not preferable in this research. From the graph, 80% (w/v) is the optimal condition because it shows the highest percentage of gel content which is 66.56%. The increment of gel content was due to crosslinking reaction occurred at higher CMSS concentration that gives the higher possibility of the CMSS to form hydrogel during the crosslinking reaction. Similar finding is stated by Nagasawa et al. [19], where the maximum percentage of the gel content is particularly dependent on the concentration of the starch. The higher the concentration of CMSS paste, the closer the CMSS macromolecules to each other to form hydrogen bonding and thus creating linkages among the CMSS macromolecules.

The degree of swelling of CMSS–acid hydrogel decreased with increase of gel content. At the highest percentage of gel content, the degree of swelling shows the lowest value which was 38.51 g/g only. The previous study also recorded that higher degree of crosslinking can reduce the swelling power [20]. When the degree of crosslinking is higher, there are more tendencies of crosslinkages to occur in the CMSS–acid hydrogel. Hence, it makes the water molecules more difficult to diffuse into the CMSS–acid hydrogel.

### Effect of acetic acid concentration

**Fig. 4:**
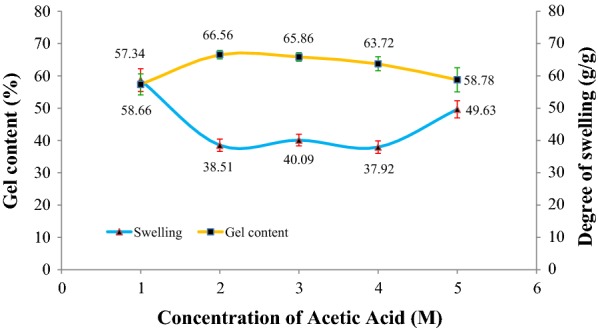
Effect of acetic acid concentration on gel content and degree of swelling of CMSS–acid hydrogel

The concentration of acetic acid was varied from 1.0 to 5.0 M as illustrated in Fig. 4. The controlled conditions for this parameter were 80% (w/v) of CMSS concentration with 24 h reaction time and 27 °C reaction temperature. At lower concentration of acid than 1.0 M, a very weak and soft hydrogel was produced that is unfavorable and not promising to be used in some applications. Figure 4 shows that there is no obvious difference in gel content for acid concentrations. The increment of gel content at 1.0 M of acetic acid to 2.0 M may be due the increment of hydrogen bonding between the CMSS–acid hydrogel molecules and it starts to decrease at 3.0 M because of acid hydrolysis reaction that disrupts the CMSS structures and the damaged chains tend to dissolve in water [21]. Thus, the gel content decreases at high concentration of acetic acid.

The degree of swelling of the CMSS–acid hydrogel is inversely proportional to the percentage of gel content. The swelling decreases from 1.0 to 4.0 M but starts to rise up to 49.63 g/g from 4.0 to 5.0 M. At high acid concentration, the acid hydrolysis onto CMSS instead of crosslinking reaction may take place to break the bond and intermolecular forces between the CMSS molecules. The breakage has interrupted the firm structure of the hydrogel itself leaving some voids to the structure and the hydrogel to absorb more water.

### Effect of time of reaction

**Fig. 5:**
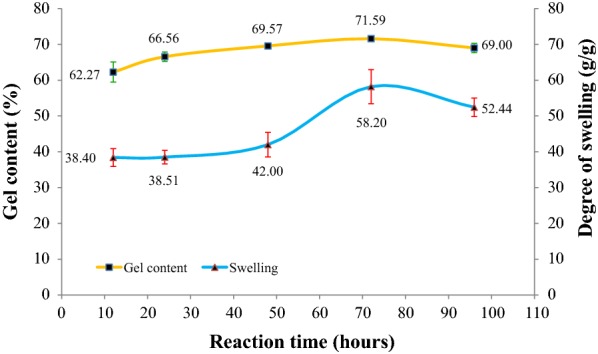
Effect of time of reaction on gel content and degree of swelling of CMSS–acid hydrogel

The effect of reaction time was studied as illustrated in Fig. 5. The reaction time varied from 12 to 96 h. CMSS concentration of 80% (w/v) with 2.0 M of acetic acid and 27 °C reaction temperature were the controlled variables for this parameter. The trend displays a gradual increment of gel content from 12 h to 72 h. The CMSS–acid hydrogel has reached its maximum gel content at 72 h of reaction which was 71.59% of gel content. The number of crosslinks increases with the increase of incubation time [22]. This will give more time of crosslinking to occur in the CMSS macromolecules. Therefore, the CMSS hydrogel attained an equilibrium within 72 h but since it was too long and more time consuming, 24 h of reaction time with 66.56% of gel content was chosen as optimum time of reaction. At 96 h of reaction, there is slight decrement of gel content due to acid hydrolysis that breaks the bond between CMSS molecules. As plotted for the degree of swelling, the trend shows that CMSS–acid hydrogel is dependent on the reaction time. As the reaction time is prolonged, the degree of swelling increases from 38.40 to 52.44%.

### Effect of reaction temperature

**Fig. 6:**
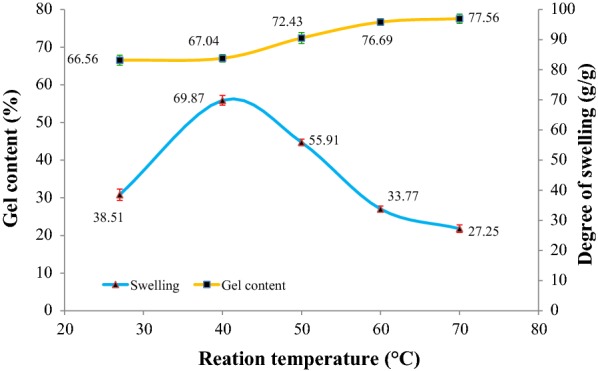
Effect of reaction temperature on gel content and degree of swelling of CMSS–acid hydrogel

Figure 6 shows the percentage of gel content and degree of swelling of CMSS–acid hydrogel at different reaction temperature. Parameters that were kept constant were 80% (w/v) of CMSS, 2.0 M acetic acid and 24 h reaction time. The CMSS–acid hydrogel is sticky and still in paste-like form at 27 °C (room temperature) and 40 °C. It started to harden and become non-sticky hydrogel as the temperature increased for more than 40 °C. The graph shows a steady increment of percentage of gel content. This is because of the extension of reaction temperature that accelerates the gel formation by promoting the formation of hydrogen bond [22]. The reaction temperature of 60 °C is chosen as the optimum since there is only a slight increment of gel content between 60 °C (76.69%) and 70 °C (77.56%).

A similar trend as observed in the previous section was found for the degree of swelling of CMSS–acid hydrogel produced at a different temperature. The degree of swelling of CMSS–acid hydrogel decreases with the increase of gel content. At higher temperature, higher possibility of formation of the hydrogen bonding that caused tighter crosslinked structure which leaves fewer voids for water absorption and thus reduces the swelling ability of the CMSS–acid hydrogel to swell in water.

### Swelling behavior in different media

**Fig. 7:**
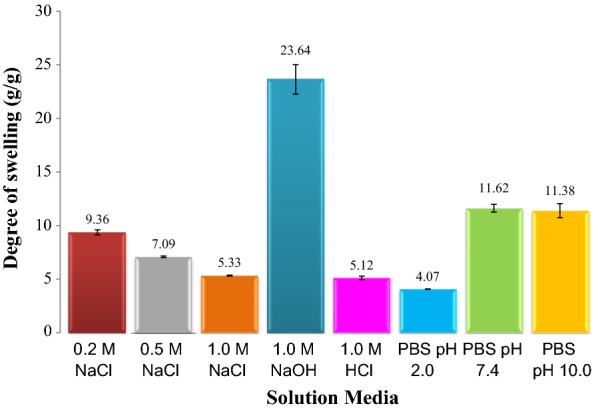
Degree of swelling of CMSS–acid hydrogel in different media

Swelling behavior of hydrogel was studied to observe the ability of the CMSS–acid hydrogel to absorb and hold some amount of water in different media either in neutral, acidic, alkaline or salt solution. The degree of swelling of the CMSS–acid hydrogel in different media is illustrated in Fig. 7. This swelling study is an important key for the future applications of the smart hydrogel. The swelling of hydrogel was conducted to study the ability of the CMSS–acid hydrogel as a smart hydrogel to swell and absorb water in various media. In addition, the special properties of these smart hydrogels are that they can either shrink or swell in any biological liquid, depending on the surrounding environment. The solution medias used were: (1) 0.2 M of NaCl solution, (2) 0.5 M of NaCl solution, (3) 1.0 M of NaCl solution, (4) 1.0 M NaOH solution, (5) 1.0 M of HCl solution, (6) PBS pH 2.0, (7) PBS pH 7.4 and (8) PBS pH 10.0.

From the optimized CMSS–acid hydrogel, the degree of swelling of CMSS–acid hydrogel in deionized water is 33.77 g/g. The first medium studied is sodium chloride (NaCl) aqueous solution. For this medium, 3 different concentrations of NaCl aqueous solution have been studied which were 0.2, 0.5 and 1.0 M. The swelling trend in NaCl aqueous solutions shows that the degree of swelling of the CMSS–acid hydrogel increases by decreasing salt concentration. The degree of swelling for 0.2, 0.5 and 1.0 M were 9.36, 7.09 and 5.33 g/g, respectively. The existence of the salt solution in the swelling medium may lead to the screening effect caused by cation (Na^+^) that leads to the osmotic pressure decrement between the CMSS–acid hydrogel and the external solution [23]. The presence of the electrolyte salt solution also causing the CMSS–acid hydrogel to not swell well due to the exo-osmosis as it tends to shrink dramatically [24]. The higher the concentration of the electrolyte salt solution, the higher the chances of hydrogel to collapse. The degree of swelling in 1.0 M of sodium hydroxide (NaOH) solution was the highest compared to other medium which is 23.64 g/g. The CMSS–acid hydrogel is an anionic hydrogel and as reported by Gupta et al. [25], the anionic hydrogel will swell in alkaline (high pH) solution. The pendant group of the anionic hydrogel, carboxyl groups, COO^−^ are ionized in higher pH level and may lead to the electrostatic repulsion and causing the swelling of the hydrogel. The next medium used was 1.0 M of hydrochloric acid (HCl) solution which gives 5.12 g/g of the degree of swelling. The negatively charged carboxyl group of CMSS–acid hydrogel react with the strong acid which cause the hydrogel to shrink, deswell and inhibit the insertion of water molecules to the hydrogel network in the acidic environment.

Meanwhile, for the phosphate buffer saline (PBS) solution, three different pH values were used to study the degree for swelling of CMSS hydrogel. The pH values are: 2.0, 7.4 and 10.0. At the lowest pH value, which is pH 2.0, the degree of swelling for the hydrogel is only 4.07 g/g. For this pH value, there is no significant difference of swelling behaviour between pH 2.0 of PBS solution and 1.0 M HCl. This is because both solutions have low pH value. Hence, it can be said that there is some interactions between the hydrogen bonding of carboxyl group of CMSS hydrogel and the PBS solution that make the hydrogel to shrink. Plus, the excess cations, H^+^ may cause the “screening effect” and the protonation of carboxymethyl group which leads to shrinkage of the CMSS–acid hydrogel [26, 27].

As the pH values of PBS increased from pH 2.0 to pH 7.4, the degree of swelling has also increased. This could be due to the transformation of COOH to COO^−^ and thus, breaking the hydrogen bonding. The breaking of hydrogen bonding led to swelling of the hydrogel. From Fig. 7, there was no obvious difference in the degree of swelling value for both PBS solutions at pH 7.4 and 10.0. The degree of swelling of CMSS–acid hydrogel slightly decreased at pH of 10.0 and similar finding was reported by Pushpamalar et al. [28].

### Fourier transform-infrared spectroscopy (FT-IR)

**Fig. 8:**
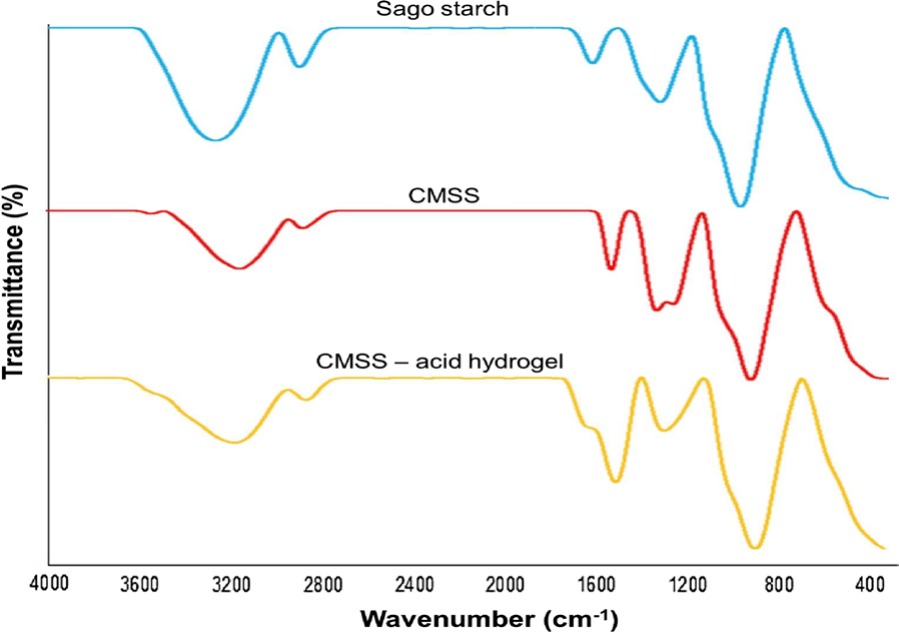
FT-IR spectra of sago starch, CMSS and CMSS–acid hydrogel

FT-IR spectra of sago starch, CMSS and CMSS–acid hydrogel are shown in Fig. 8. For sago starch IR spectrum, a strong absorption band at 3273.20 cm^−1^ which indicated the presence of hydroxyl group of polysaccharide chain, –OH stretching vibration, as well as intramolecular and intermolecular hydrogen bonds in the glycosidic bond in the sago starch molecule [29]. Meanwhile, at 1350.40 cm^−1^, this absorption band was due to the existence of the alkane group but with a different type of vibration, –CH bending vibration. For IR spectrum of CMSS, the broad absorption band at 3175.19 cm^−1^ was less intense compared to sago starch. This is due to the substitution of COONa replacing the –OH group. There was a new absorption band at 1596.59 cm^−1^ which attributed to the substitution of COO^−^ group into the sago starch. Jamingan et al. [29] also reported a similar result which affirmed the carboxymethylation has taken place on the starch molecules. However for CMSS–acid hydrogel, there was an additional shoulder band at 1720.73 cm^−1^ that showed the functional group of carboxyl which is C=O bond vibration [30]. This band confirmed the presence of carboxylic acid, due to the reaction of COO^−^ in the CMSS with H^+^ from the acetic acid.

### X-ray diffraction (XRD)

**Fig. 9:**
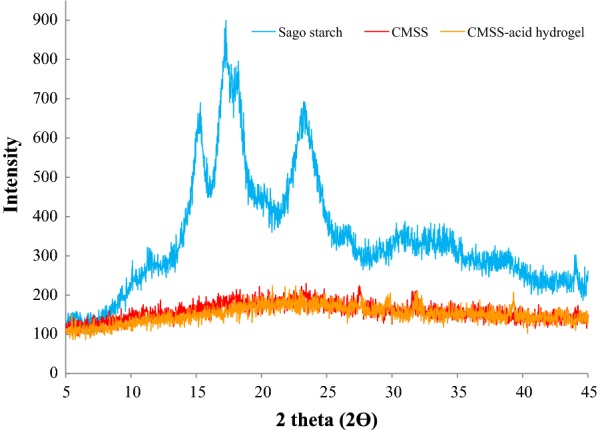
XRD diffractogram of sago starch, CMSS and CMSS–acid hydrogel

From the diffractogram in Fig. 9, sago starch has C-type diffraction pattern which is a mixture of A-(65%) and B-(35%) types. Sago starch has broad and strong diffraction peaks at 15.32°, 17.28°, 18.22° and 23.32° which confirmed its semi-crystalline nature. The peaks observed were broad due to the small crystallites of the sago starch. These results are also in agreement with study conducted by Rachtanapun and Simasatitkul [31].

The diffractograms of CMSS and CMSS–acid hydrogel, showed only broad patterns which attributed to the amorphous phase. This has confirmed that both samples lose their crystallinity, which may be due to the replacement of hydroxyl groups in the samples [32] and the breakage of starch due to heat with presence of water [33].

The loss of crystalline phase in sago starch can be seen after the modification to both CMSS and CMSS–acid hydrogel. This was due to the presence of strong alkaline, NaOH during carboxymethylation that transformed the hydroxyl groups of starch molecules (St-OH) into alkoxide group (St-O^−^). Effects from the repulsion of both negative charges caused a tension on neighbouring crystallites of starch molecule which pointed to the dissociation of double-helical regions and the disintegration of the crystalline structure [34].

### Scanning electron microscopy (SEM)

**Fig. 10:**
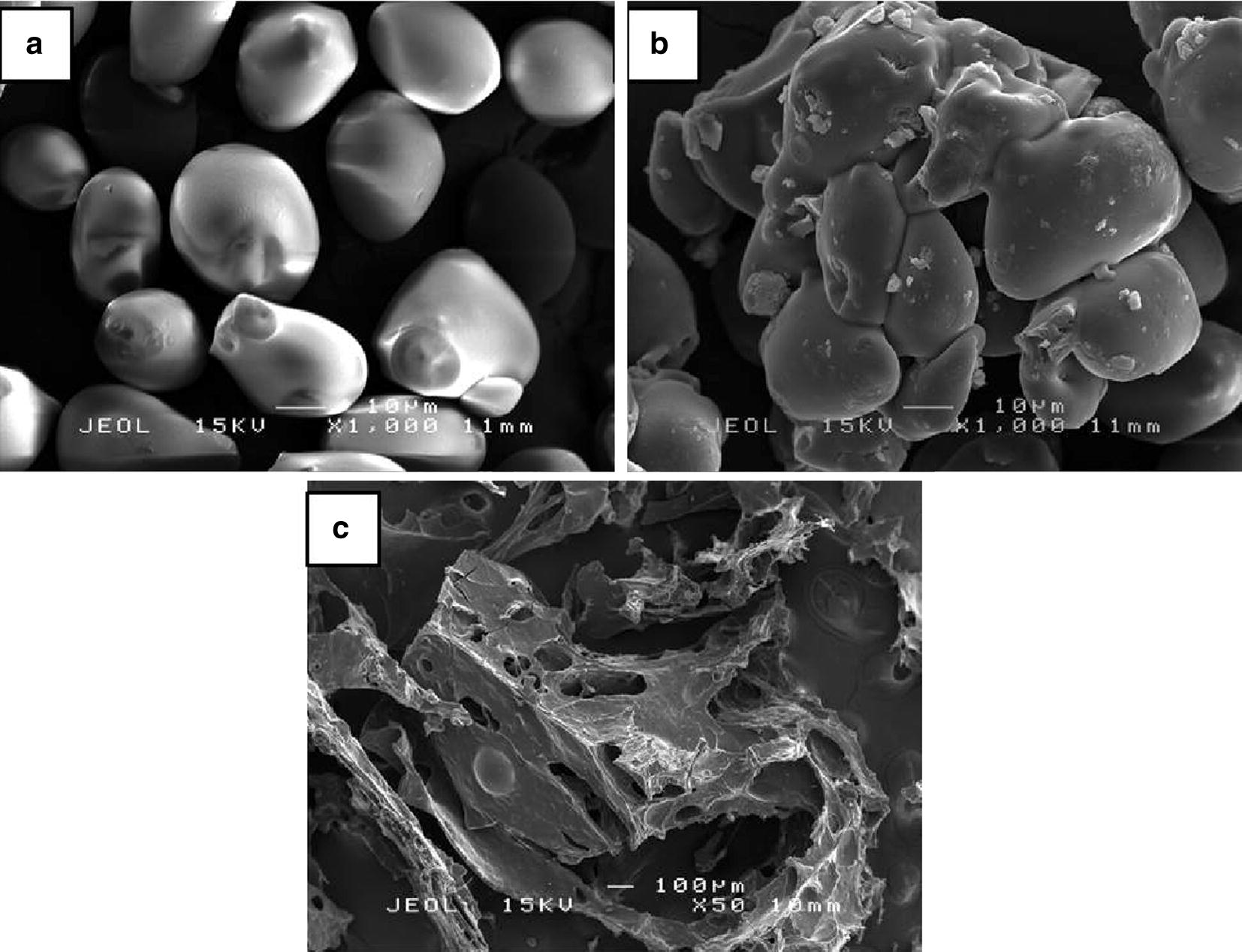
Scanning electron micrograph of **a** sago starch, **b** CMSS and **c** CMSS–acid hydrogel

Figure 10a shows that the sago starch granules are in oval ‘egg-shaped’ with some curtailed side. The sago starch granules have smooth, fine and unwrinkled surface and the diameter of oval granules of sago starch is estimated in the range of relatively 20–50 µm similar to the previous study reported by Uthumporn et al. [35] and Ahmad et al. [36]. Figure 10b obviously exhibits an irregular shape of CMSS granules. The CMSS lost its smooth exterior by having a rough and groove surface which was recognized on the modified sago starch granules and this is due to the loss of crystalline structure as stated by Basri et al. [15]. The micrograph shows that the CMSS remain undamaged despite been through the modification with SMCA and some alcoholic solvents. Figure 10c shows the morphology of CMSS–acid hydrogel at magnifications of 50×. SEM was used to examine the morphology and porosity of the CMSS–acid hydrogel after it was crosslinked through hydrogen bonding. At 50× magnification, it shows the overall pores of the CMSS–acid hydrogel and the erratic and irregular pores of the hydrogel can clearly be seen.

## Conclusions

In this study, CMSS was modified to obtain the CMSS–acid hydrogel. The preparation of the CMSS–acid hydrogel was successfully optimized with all parameters studied. Swelling in a different media of the hydrogel shows that the CMSS–acid hydrogel is a smart hydrogel that change its behavior depending on the surrounding behavior. The CMSS–acid hydrogel swells in both alkaline and salt solution but will shrink in acidic solution. Due to these smart properties of the CMSS–acid hydrogel, it can be used in various industrial applications.
